# Molecular Forcefield Methods for Describing Energetic Molecular Crystals: A Review

**DOI:** 10.3390/molecules27051611

**Published:** 2022-02-28

**Authors:** Wen Qian, Xianggui Xue, Jian Liu, Chaoyang Zhang

**Affiliations:** 1 Institute of Chemical Materials, China Academy of Engineering Physics, Mianyang 621999, China; qianw03@caep.cn (W.Q.); xuexg@caep.cn (X.X.); liujian-12@caep.cn (J.L.); 2 Beijing Computational Science Research Center, Beijing 100048, China

**Keywords:** molecular forcefield, energetic molecular crystal, molecular dynamics simulation, ReaxFF

## Abstract

Energetic molecular crystals are widely applied for military and civilian purposes, and molecular forcefields (FF) are indispensable for treating the microscopic issues therein. This article reviews the three types of molecular FFs that are applied widely for describing energetic crystals—classic FFs, consistent FFs, and reactive FFs (ReaxFF). The basic principle of each type of FF is briefed and compared, with the application introduced, predicting polymorph, morphology, thermodynamics, vibration spectra, thermal property, mechanics, and reactivity. Finally, the advantages and disadvantages of these FFs are summarized, and some directions of future development are suggested.

## 1. Introduction

Energetic materials (EM) act as the key chemical activity component in weapon systems, and energetic crystals are the main and functional part therein [1]. Until now, the most widely used energetic crystals have been molecular crystals, including TNT, RDX, HMX, and TATB. The thermal properties, mechanical properties, and chemical reactivity of energetic molecular crystals are of great importance for a whole EM [2]. These properties can be measured in experimental ways, such as thermal analysis, mechanical tests, detonation measurements, and sensitivity tests [3]. However, because of the particular needs for test samples and risk in explosive tests, not all the properties can be obtained from experiments. With the development of quantum chemistry, molecular simulation, algorithms, and computer technology, theoretical methods play an important part in investigating the properties [4,5]. The molecular modeling-based theoretical methods can effectively describe the inter-molecular interactions in molecular crystals—including van der Waals forces, electrostatic interactions, and hydrogen-bonding [6,7,8,9,10]—as the driving force for molecular stacking. Thus, they can provide an efficient way to predict crystalline structures and properties precisely, so as to construct quantitative structure–property relationships (QSPRs) [11,12,13]. 

Molecular dynamics is a basic means of molecular simulation, and describes the movement of the particles in a particular system, based on the second Newton’s law [14]. Ensemble is used to simulate the environment of a multi-particle system, such as NVE with constant particle number, energy, and volume; NVT with constant temperature, volume, and temperature; NPT with constant particle number, pressure, and temperature, etc. [15]. The thermostat and barostat are necessary to control the temperature and pressure, respectively [16,17,18]. Periodic boundary conditions (PBCs) [19] are applied for condensed state systems.

**Figure 1:**
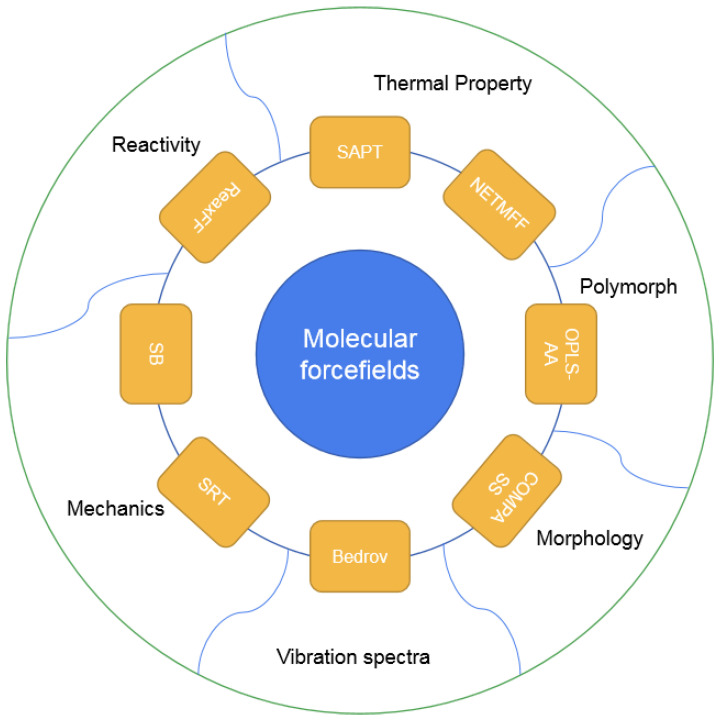
Typical molecular FFs applied for energetic crystals and their main applications.

A popular method of MD simulation for molecular materials is the use of molecular forcefields (FFs), including classic FFs, consistent FFs, and reactive FFs. They have been applied for energetic crystals, the details are as shown in Figure 1. Molecular FF methods can be utilized to predict polymorph, morphology, vibration spectra, thermal properties, mechanical properties, and reactivity related to detonation and sensitivity for various energetic molecular crystals. This paper reviews the principles, applications, and comparisons of these commonly used FF methods for EMs, aiming to provide an efficient solution for predicting structures and properties for energetic molecular crystals in particular application situations.

## 2. Classic Forcefield Refitted for EMs and Their Applications

### 2.1. Development of Refitted FFs

**Figure 2:**
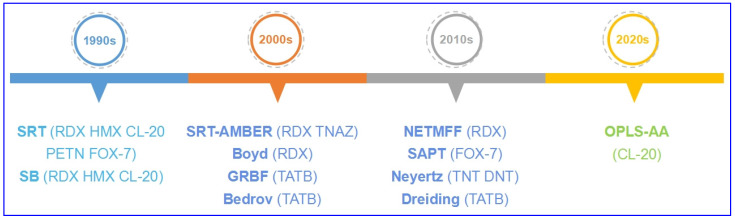
Development of classic FFs refitted for EMs.

In order to investigate the precise structure and properties of energetic molecular crystals, classic molecular FFs were refitted for EMs (Figure 2 and Table 1). In the 1990s, Sorescu et al. [20,21,22,23,24,25] developed a classic FF (SRT) with pair potential refitted for predicting lattice parameters, density, bulk modulus, and shear modulus of aliphatic energetic compounds—including nitramine heterocycles RDX, HMX, and CL-20, and linear conjugated energetic compound FOX-7. It was also applied on linear nitrate PETN which has overloaded substituents with lower accuracy [26]. Smith et al. [27] developed another kind of multi-particle potential, the Smith–Bharadwaj (SB) potential, which is widely used in the calculations of shock compression, shear bands, elastic constants, and modulus for HMX, CL-20, and RDX [28,29,30,31]. 

In the 2000s, more classic FFs were applied. For RDX crystal, Agrawal et al. [32,33] combined the advantages of traditional AMBER FF with SRT FF, and applied the refitted FF (SRT-AMBER) which can effectively describe the flexibility of molecules on the calculations of cell parameters, melting point, and mechanics for TNAZ and RDX. Boyd et al. [34] also developed a multi-particle potential for RDX; thereby, the vibration spectra, thermodynamics, thermal expansion, and mechanics were calculated. For aromatic nitro compound TATB, a multi-particle FF (GRBF) with Lennard–Jones portion is developed by Gee et al. [35] using an ab initio approach for large molecular system TATB crystal, with cell parameters, density, thermal expansion, and pressure–volume isotherm prediction. Bedrov et al. [36] developed a potential to calculate the vibration spectra, elastic stiffness coefficients, and isotropic moduli; moreover, thermal conductivity can also be obtained using non-equilibrium molecular dynamics (NEMD) under Bedrov’s potential [37].

In the 2010s, the symmetry-adapted perturbation theory (SAPT)-based potential was also applied to describe thermal properties for FOX-7 [38], and thermal expansion and pressure response were investigated thereby. Neyertz et al. [39] developed a classic potential for aromatic nitro compounds TNT and DNT, which possess conjugated structure, and calculated the cell parameters, density, tensile modulus, bulk modulus, and shear modulus. Song et al. [40] developed an all-atom, non-empirical, tailor-made FF (NETMFF) for RDX, and cell parameters, density, and thermal expansion were predicted thereby; the Dreiding FF was also applied on TATB, with the anisotropic thermal expansion simulated successfully [41,42].

**Table 1:** Energy expressions and application scopes for the classic FFs.

FFs	Valence Terms	van der Waals Interaction Term	Electrostatic Interaction Term	Applications
SRT [20]	\	Buckingham 6-exp form	Coulomb function	RDX HMX CL-20 FOX-7 PETN (lattice parameter, density, mechanics)
SAPT [38]	\	Buckingham 6-exp form	Simplified Coulomb function	FOX-7 (thermal properties, pressure responses, isothermo)
SRT-AMBER [32]	Harmonic bond stretching, harmonic angle bending, cosine torsion term	Buckingham 6-exp form	Coulomb function	RDX (lattice parameter, density, melting point, mechanics) TNAZ (lattice parameter, density, melting point)
SB [27]	Harmonic bond term, angle term, dihedral term, anharmonic torsion term	Buckingham form	Coulomb function	RDX HMX CL-20 (shock compression, shear bands, elastic constants and modulus)
Boyd’s [34]	Bond stretching described by Morse function, angle bending described by harmonic function	Buckingham LJ 6-12 form	Coulomb function	RDX (lattice parameter, density, thermodynamics, vibration spectra, thermal expansion, mechanics)
NETMFF [40]	Bond term, angle term, dihedral (torsion angle) term, out-of-plane bending angle term, cross-coupling terms of bond–bond, bond–angle couplings	Damped Buckingham form	Coulomb function	RDX (lattice parameter, density, thermal expansion)
GRBF [35]	Harmonic bond stretch term, bond-angle bend term, dihedral angle torsion term	Lennard–Jones 12-6 form	Coulomb function	TATB (lattice parameters, density, thermal expansion, isotherm)
Bedrov’s [36]	Harmonic functions of covalent bonds, three-center bends, and improper dihedrals	Buckingham 6-exp form	Coulomb function	TATB (lattice parameter, thermal expansion, mechanics, vibration spectra, thermal conductivity)
Neyertz’s [39]	Angle-bending deformations described by harmonic function, torsional motions around the dihedral angles τ, sp^2^ ring and NO_2_ structures kept planar described by harmonic function	Lenard-Jones 12-6 form	Coulomb function	TNT DNT (lattice parameter, density, tensile, bulk and shear modulus)
Dreiding [41]	Bond stretching interaction term, angle bending interaction term, dihedral angle interaction term, inversion interaction term	Lennard–Jones 12-6 form	Coulomb function	TATB (geometries, crystal packing, thermal expansion)
OPLS-AA [43]	Bond term, angle term, dihedral term	Lennard–Jones 12-6 form	Coulomb function	CL-20 (lattice parameter, density, polymorph prediction)

In 2021, Wang et al. developed a tailor-made OPLS all-atom FF (OPLS-AA) [43] with polymorphism of CL-20 being predicted successfully [44].

### 2.2. Functional Forms of the Refitted FFs

The energy expressions of the classic FFs have something in common, i.e., most of FFs have valence terms, van der Waals interaction terms, and electrostatic interaction terms, and they differ only in functional forms (Table 1). For aliphatic energetic compounds including heterocycles RDX, HMX, CL-20, and linear PETN, FOX-7, SRT potential was applied. The energy expression of SRT only has a pairwise Buckingham 6-exp form with repulsion and dispersion interactions, and a Coulombic potential of electrostatic interactions [20]. The energy expression of SAPT potential is similar to the SRT pair potential, and it was also applied on FOX-7 [38].

For the multi-particle potentials of nitramine heterocycles, the energy terms of SB potential cover harmonic bonds, angles, dihedrals terms, anharmonic torsions, and non-bonded Buckingham and Coulomb interactions [27]. SRT-AMBER combines the forms of SRT and AMBER FFs, with harmonic bond stretching and angle bending terms, cosine torsion term, and nonbond term replaced by Buckingham (exp-6) potential and Coulombic potential in SRT [32]. Boyd’s multi-particle potential contains bond stretching, angle bending, vdW term, and electrostatic term; described by Morse function, harmonic function, Buckingham potential LJ 6-12, and Coulomb function, respectively [34]. In comparison, NETMFF is more complex, with more valence terms included, as listed in Table 1 [40]. 

For the aromatic nitro compounds, GRBF and Bedrov’s potential were applied for TATB, and Neyertz’s FF was applied for TNT and DNT. GRBF FF includes valence terms containing harmonic bond stretch, bond-angle bend, dihedral angle torsion, and intermolecular pair potentials, including a Coulombic electrostatic term *E*_el_ and a Lennard–Jones LJ 12-6 term [35]. Bedrov’s potential includes the harmonic functions of covalent bonds, three-center bends and improper dihedrals, and nonbonded interactions of Buckingham (exp-6) potential and Coulomb interactions [36]. In Neyertz’s potential, angle-bending deformations are described by harmonic function, and torsional motions around the dihedral angles τ are represented by a polynomial in cos τ, while sp^2^ ring and NO_2_ structures maintain planarity by using harmonic function [39]. Moreover, some general FFs were also refitted for energetic crystals, such as Dreiding for TATB [41] and OPLS-AA for CL-20 [43]. 

To date, the effects of different molecular structures—especially functional groups—were considered to make precise predictions. Initially, SRT [20] and SAPT FFs [38] are simple pair potentials which only describe the intermolecular interactions, with assumption of rigid molecules, thus they cannot describe the effect of different molecular structures, resulting in lower accuracy. Combing the rigid-molecule SRT potential with intramolecular interactions from AMBER, the SRT-AMBER potential [32] accurately predicted the chair and inverted chair conformation, bond lengths, and bond angles of the RDX molecule; however, the parameters for the N-NO_2_ improper dihedral angles are not available in AMBER, and the parameters for O-NO_2_ were used in SRT-AMBER, and there are some inaccuracies in the calculated orientations of the NO_2_ groups; thus, modifications in the torsional parameters are needed. SB potential [27] with anharmonic torsions terms that display extrema at the torsion angles that correspond to stationary points on the conformational energy surface can effectively predict lattice parameters and elastic tensors for RDX and HMX. Furthermore, Boyd’s potential [34] managed to stabilize the RDX crystal lattice with flexible molecules in the correct conformation, with Morse bond stretching, harmonic angle bending, cosine torsions terms, and in which the torsion parameter C-N-N-O values were modified to adjust the N-NO_2_ rotational barrier. Moreover, in NETMFF for RDX [40], many more parameters regarding functional groups were considered, including parameters c_4 for the carbon atom, n_3r for the nitrogen atom of the triazine ring, n_3o for the nitrogen atom of the NO_2_ group, o_1 for the oxygen atom, and h_1 for the hydrogen atom; as well as torsion parameters for six RDX conformers. Consequently, the angles and torsions for the NO_2_ group are well described, and the lattice parameters and thermal expansion are well predicted. In GRBF for TATB [35], the amino C-C-N-H and nitro C-C-N-O group rotational barriers were modified, and the nonbonded interactions for amino groups and nitro groups were also considered; similar parameters were considered in Neyertz’s potential for TNT and DNT [39], as well as in Bedrov’s [36] and Dreiding potentials for TATB [41]. The differences in the functional forms, including descriptions of functional groups for the FFs, bring differences in prediction precision and areas of application.

### 2.3. Application of Prediction

#### 2.3.1. Cell Parameters and Density

The refitted FFs can effectively predict cell parameters, and further density. For example, the cell parameter and density of RDX have been separately predicted using SRT, SRT-AMBER, Boyd’s FF, SB, and NETMFF potentials [20,27,32,34,40]. In comparison, the density prediction by NETMFF is much closer to the experimental results [45], while those by SRT and SRT-AMBER are not so accurate. It is attributed to SRT’s lack of valence terms, while NETMFF has the greatest abundance of valence terms. It shows that the valence terms are important in describing the structural properties of cell parameters and density. Furthermore, the cell parameters and density of TATB can be described well by GRBF, Bedrov’s, and Dreiding FFs [34,35,42]; and those of TNT, DNT, and CL-20 can be described well by Neyertz’s FF [39], and SB [27] and OPLS-AA FFs [44], respectively.

#### 2.3.2. Polymorphism

Classic FFs have been refitted to predict various crystalline phases and their properties. For example, the Neyertz’s FF [39] was applied to monoclinic and orthorhombic TNT, with density, tensile modulus, bulk modulus, and shear modulus predicted, in agreement with experimental results. Wang et al. [44] also applied the OPLS-AA FF for the polymorph prediction of CL-20, in which the FF parameters were refitted based on DFT calculations with corrected density functional M06-2X, with most polymorphs being reproduced successfully. 

#### 2.3.3. Vibration Spectra

Kroonblawd et al. [37] used the modified Bedrov’s potential to investigate the vibration spectra of TATB. The modified FF overcame the gap between the prediction by the original one and experimental observations. The calculated spectrum can effectively assign the vibration modes, including amine antisymmetric stretch, amine symmetric stretch, amine scissoring plus C-NH_2_ stretch, nitro antisymmetric stretch plus amine scissor/rock, and ring stretch. The Boyd’s potential [34] has also been effectively applied to investigating the vibration spectra for RDX, in which special attention has been paid to the vibrational states between 200 and 700 cm^−1^ described as “doorway modes” for the transfer of energy from lattice phonons to the molecular vibrations involved in bond breaking [46,47].

#### 2.3.4. Thermal Property

Among the thermal properties, thermal expansion is an important feature related to performance. The refitted FFs effectively described the linear and volume thermal expansion for series of energetic crystals: the SRT FF has been refitted for FOX-7, and the linear and volume thermal expansion coefficients (CTE) of FOX-7 crystal were determined from the averages of lattice dimensions extracted from trajectories of MD calculations; the results of linear CTE values show high anisotropy because of the layer wave-like stacking style inside the FOX-7 crystal, in agreement with experiment [26]. The MD simulations for CTE of FOX-7 were also carried out with the fitted SAPT potential, showing a significant anisotropy along different crystalline directions [38]. Compared with experiment [48], SRT is better than SAPT, since SRT and SAPT have the same vdW term, while SAPT has a simplified Coulomb term. It demonstrates the importance of electrostatic interaction in the thermal expansion of FOX-7 crystal. Furthermore, SRT, SRT-AMBER, Boyd’s, and NETMFF FFs were refitted for thermal expansion of RDX crystal. Compared with the experiments [48], it was found that SRT and Boyd’s FFs describe the anisotropy better, while NETMFF tends to isotropy [20,27,34,40]. The GRBF FF [35] was used to predict the CTE for TATB, agreeing well with the experiments [49]. Dreiding FF was also applied to the TATB crystal, with the planar stacking maintained and the anisotropic thermal expansion reproduced successfully [41,42]. Thermal conductivity of TATB was calculated using NEMD with Bedrov’s potential [36]. Therein, the thermal flux, temperature gradient, as well as resulting thermal conductivity, were obtained from direct MD simulations of the slabs, the results from different directions show high anisotropy [37].

#### 2.3.5. Mechanical Property

The SRT pair potential was refitted for RDX using quantum chemistry calculations at the theoretical level of MP2, and NPT-MD with this refitted potential were performed to predict mechanical properties—including bulk modulus and volume compressibility which match with experiments [20]. The SRT-AMBER potential has also been used to investigate the mechanics for crystalline RDX; however, the prediction accuracy of SRT-AMBER has no advantages compared with other FFs, thus the FF parameters need further modification [32]. Additionally, the SB potential has widely been used to calculate the mechanical properties for energetic crystals of HMX, CL-20, and RDX, with elastic constants and bulk modulus being obtained [27]; the Neyertz’s potential was applied to predict the tensile modulus, bulk modulus, and shear modulus for TNT and DNT [39]; and the elastic stiffness coefficient, bulk modulus, and shear modulus of TATB were accurately predicted using Bedrov’s potential [36,37].

#### 2.3.6. Shock Responses

**Figure 3:**
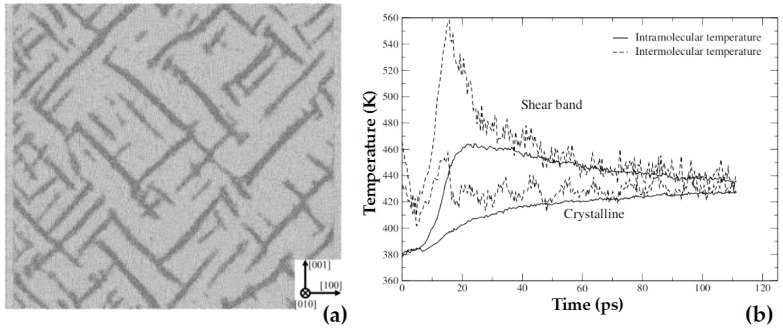
Shock-induced shear bands (**a**) and the intermolecular and intramolecular temperatures (**b**) in RDX crystal calculated using SB potential. Reprinted by permission from [28]; copyright 2008 American Physical Society. Permission conveyed through the American Physical Society and SciPris.

For shock responses, the SB potential can effectively describe the shock-induced shear bands in RDX crystal, in which the intermolecular and intramolecular temperatures of a crystalline region and adjacent shear band can be calculated (Figure 3) [28]. Moreover, it can describe the shock compression in the RDX crystal, including stress along compression direction, temperature evolution under shock compression, compression ratio, and temperature as a function of shock pressure [29].

## 3. Consistent Forcefields and Their Applications for EMs

### 3.1. Theory

Apart from the FFs refitted for energetic compounds, some of the consistent FFs have also been applied in energetic crystals. The consistent FFs with more complicated energy terms are parameterized from large numbers of experimental data of organic compounds and other species, and they are more precise compared with the universal FFs [50,51,52,53,54,55,56]. The consistent FFs include classic CFF series [57,58,59,60,61], commonly used PCFF (the polymer consistent force field) [62,63,64] and COMPASS (condensed-phase optimized molecular potentials for atomistic simulation studies) [65,66,67,68]. PCFF and COMPASS are more advanced and share the same functional form, in which the analytical expression of energy surface including four-order function form of bond stretching (bond); four-order function form of angle bending (angle); dihedral angle term (dihedral); out-of-plane term (OOPA); the mixing terms of bond, angle, and torsion (bond/bond, bond/angle, angle/angle, bond/torsion, angle/torsion, angle/bond/torsion); and the non-bond interaction term (nonbond) shown as Equation (1).

(1) $$\begin{array}{l} E=\sum_{\mathrm{bond}}\left[K_{b2}{\left(b-b_{0}\right)}^{2}+K_{b3}{\left(b-b_{0}\right)}^{3}+K_{b4}{\left(b-b_{0}\right)}^{4}\right]+\\ \sum_{\mathrm{angle}}\left[K_{a2}{\left(\theta -\theta_{0}\right)}^{2}+K_{a3}{\left(\theta -\theta_{0}\right)}^{3}+K_{a4}{\left(\theta -\theta_{0}\right)}^{4}\right]+\\ \sum_{\mathrm{dihedral}}\left[K_{t1}(1-\cos\phi )+K_{t2}(1-\cos2\phi )+K_{t3}(1-\cos3\phi )\right]+\\ \sum_{\mathrm{OOPA}}K_{\chi }{\left(\chi -\chi_{0}\right)}^{2}+\sum_{\mathrm{bond}/\mathrm{bond}}K_{bb}(b-b_{0})(b^{\prime }-b_{0}^{\prime })+\\ \sum_{\mathrm{bond}/\mathrm{angle}}K_{ba}(b-b_{0})(\theta -\theta_{0})+\sum_{\mathrm{angle}/\mathrm{angle}}K_{aa}(\theta -\theta_{0})(\theta^{\prime }-\theta_{0}^{\prime })+\\ \sum_{\mathrm{bond}/\mathrm{torsion}}\left(b-b_{0})(K_{bt1}\cos\phi +K_{bt2}\cos2\phi +K_{bt3}\cos3\phi \right)+\\ \sum_{\mathrm{angle}/\mathrm{torsion}}\left(\theta -\theta_{0})(K_{at1}\cos\phi +K_{at2}\cos2\phi +K_{at3}\cos3\phi \right)+\\ \sum_{\mathrm{angle}/\mathrm{torsion}/\mathrm{angle}}k(\theta -\theta_{0})(\theta^{\prime }-\theta_{0}^{\prime })(\phi -\phi_{0})+\\ \sum_{\mathrm{nonbond}}\left\{\varepsilon_{ij}[2{\left(\frac{r_{ij}^{0}}{r_{ij}}\right)}^{9}-3{\left(\frac{r_{ij}^{0}}{r_{ij}}\right)}^{6}]+\frac{q_{i}q_{j}}{r_{ij}}\right\} \end{array}$$

where *b* is the bond, *θ* is the angle, *ø* is the torsion angle, and *χ* is the out-of-plane angle; *K_b_*, *K_a_*, *K_t_*, *K_χ_*, *K_bb_*, *K_ba_*, *K_aa_*, *K_bt_*, *K_at_*, *k* are the fitting parameters for bond, angle, dihedral, OOPA, and mixing terms, respectively; *r_ij_* is the distance between two valence-bonded atoms *i* and *j*, *q_i_* and *q_j_* are their electronic charges, and *ε_ij_* and *r*_0_ are the LJ-9-6 parameters. It can be noticed that, in the non-bond interactions, the van der Waals (vdW) interaction term is in the form of Lennard–Jones (LJ) 9-6, for which the mixing rule is shown as Equations (2) and (3), where *ε_i_* and *ε_j_* are the fitting parameters.

(2) $$r_{ij}^{0}={\left[\frac{{\left(r_{i}^{0}\right)}^{6}+{\left(r_{j}^{0}\right)}^{6}}{2}\right]}^{1/6}$$

(3) $$\varepsilon_{ij}=2\sqrt{\varepsilon_{i}\varepsilon_{j}}\left[\frac{{\left(r_{i}^{0}\right)}^{3}{\left(r_{j}^{0}\right)}^{3}}{{\left(r_{i}^{0}\right)}^{6}+{\left(r_{j}^{0}\right)}^{6}}\right]$$

The above functional form (Equations (1)–(3)) applied for both the PCFF and COMPASS, differing mainly in the range of functional groups to which they were parameterized, as well as combination rules for non-bond terms. To date, the COMPASS FF was developed based on the CFF series and PCFF, with many more cross-items in the energy expression to improve the accuracy of the calculations. In addition, COMPASS was parameterized and validated for organic compounds which contain nitro and azide groups, thus it is applied well on energetic compounds including RDX, HMX, CL-20, FOX-7, TATB, TNT, and others. The consistent FFs were directly used in the MD simulations without any refitting.

### 3.2. Applications

#### 3.2.1. Morphology

Crystal morphology is of significance because it will affect the properties and performances of energetic crystals even with the same molecular formula or crystalline structure. Theoretical models, such as attachment energy model (AE) [69,70] and Bravais–Friedel–Donnay–Harker model (BFDH) [71,72,73,74], combined with MD simulations, were used to predict various morphologies of energetic crystals under the effects of solvent, temperature, pressure, and so on. For example, the crystal morphologies of HMX, RDX, and insensitive energetic crystal TATB were efficiently predicted using BFDH model and MD simulations [75,76,77,78].

Considering the solvent effect, the crystalline growth patterns and morphology for nitroamino explosives of RDX, β-HMX, and ε-CL-20 in various solvents were predicted using MD simulations combined with consistent FF COMPASS. It was found that different solvents may cause different morphologies for each energetic crystal [79,80]. Song et al. [81] applied the AE model and MD simulations to simulate the morphology of DNP grown from different solvents. Zhu et al. [82] used MD simulations and a modified AE model with solvent effect considered to predict the morphology of the MTNP/CL-20 cocrystal, which was consistent with experimental observations.

The morphology prediction based on the AE model features higher accuracy and is widely used with considering solvent effects; while the BFDH model shows higher efficiency. In fact, BFDH is so fast that it can handle a large amount of crystals in a relatively very short time, which can be considered in the high-throughput computation.

#### 3.2.2. Polymorphism

The consistent FFs were also employed to predict the polymorphism for energetic crystals. For example, the polymorphs of CL-20 were studied using COMPASS [83], the possible space groups of P_21/c_, P-1, P_212121_, P_21_, P_bca_, C_2/c_, and P_na21_ were investigated. The polymorphs of a series of compounds of polynitrohexaazaadmantanes were predicted, including 2,4,6,8-tetranitrohexaazaadmantane, 2,4,6,8,10-pentanitrohexaazaadmantane, and 2,4,6,8,9,10-hexanitrohexaazaadmantane, with results close to the experimental observations [84].

#### 3.2.3. Properties 

Thermodynamic properties are crucial to energetic crystals. For example, COMPASS-MD simulations were used to compare the total energies for α/γ, β, and ε-formed conformations of CL-20 with or without solvents; as a result, β-CL-20 is the most energetically favored and easily converted into ε-CL-20 crystal [85]. COMPASS was also employed to calculate lattice energy for a series of co-crystals involving CL-20, HMX, TNT, BTF, TNB, TNA, MATNB, or TNAZ molecules [86]. Intermolecular interaction energy between energetic crystals and their additives can also be described well using COMPASS [75,76,77,78]. Moreover, COMPASS was utilized in the prediction of thermal expansion for energetic crystals—including β-HMX, FOX-7, TNAD and bicyclo-HMX—in which lattice parameters as a function of temperatures were computed, and linear or volume CTEs were obtained [42,87,88,89].

COMPASS was also utilized to study mechanical properties for various energetic crystals, with elastic constant tensor and Hooke’s Law [90,91]. For example, the Young’s modulus, bulk modulus, shear modulus, and Poisson’s ratio of RDX, HMX, and CL-20 crystals have been obtained using COMPASS FF based MD simulations under NPT ensemble [92,93,94,95]. The Young’s modulus and Possion’s ratio of newly synthesized energetic crystals α-DATNBI and β-DATNBI were also calculated using MD simulations and mechanical computations, with results showing that the mechanical properties of β-DATNBI are closer to β-HMX and better than α-DATNBI [96]. It should be noticed that mechanical calculations using COMPASS-MD have been applied for some of the nitro compounds or nitroamine explosives only, while rarely being applied for other EMs. The COMPASS-MD results were also analyzed to evaluate thermal stability and sensitivity [95]. COMPASS is efficient at predicting the morphology of energetic crystals, and thereby calculating properties including thermodynamics, thermal expansion, mechanical properties, and stability. In general, the refitted FFs can present higher accuracy in contrast to the consistent ones.

## 4. Reactive Forcefields and the Applications for EMs

### 4.1. Theory

Reactivity is the key property for energetic crystals, which is highly related to the detonation and sensitivity of EMs. Theoretical methods—including Reactive FFs [97,98,99,100], AIMD [101,102,103,104,105,106,107], and DFTB-MD [108,109,110,111,112,113,114]—were utilized to reveal reaction mechanism of energetic crystals, and ReaxFF is the most efficient. The reactive FF is a unique type of molecular FF, which can describe not only the interactions between particles and their movements, but also the chemical bond formation and rapture, namely chemical reactions, in the process. Reactive FF began with REBO FF with Abell–Tersoff function form [97], while the most widely used one is ReaxFF developed by Goddard’s research group [98,99,100]. ReaxFF contains combined parameters for large number of organic and inorganic systems, with an energy expression shown as Equation (4).

(4) $$\begin{array}{l} E_{\mathrm{Reax}}=E_{\mathrm{bond}}+E_{\mathrm{lp}}+E_{\mathrm{over}}+E_{\mathrm{under}}+E_{\mathrm{val}}+E_{\mathrm{open}}\\ +E_{\mathrm{coa}}+E_{\mathrm{tors}}+E_{\mathrm{conj}}+E_{\mathrm{Hbond}}+E_{\mathrm{vdW}}+E_{\mathrm{coulomb}} \end{array}$$

where the total energy of system (*E*_Reax_) includes bond dissociation energy term (*E*_bond_), lone pair energy penalty term (*E*_lp_), energy penalty for over-coordinated atoms (*E*_over_), energy contribution for the resonance of the n-electron between attached under-coordinated atomic centers (*E*_under_), angle bending energy term (*E*_val_), energy penalty needed to reproduce the stability of systems with double bonds sharing an atom in a valency angle (*E*_pen_), three-body conjugation term to describe the stability of -NO_2_ groups (*E*_coa_), torsion angle energy term (*E*_tors_), contribution of conjugation effects to the molecular energy (*E*_conj_), H-bond energy term (*E*_H-bond_), van der Waals interaction term (*E*_vdW_), and Coulomb interaction term (*E*_Coulomb_). In addition, long-range London dispersion (*E*_lg_) correction was added in the total energy terms of ReaxFF-lg (*E*_Reax-lg_) [115], where *r_ij_* is the distance between atom *i* and atom *j*, *R_eij_* is the equilibrium vdW distance between atoms *i* and *j*, and *C_lg,ij_* is the dispersion energy correction parameter (Equations (5) and (6)).

(5) $$E_{\mathrm{Reax}-\mathrm{lg}}=E_{\mathrm{Reax}}+E_{\mathrm{lg}}$$

(6) $$E_{\mathrm{lg}}=-\sum_{ij,i<j}^{N}\frac{C_{\mathrm{lg},ij}}{r_{ij}{}^{6}+dR_{eij}{}^{6}}$$

To date, the key of ReaxFF is the calculation of Bond order (*BO_ij_*), which can be derived from the calculations of σ-bonds (*BO_ij_^σ^*), π-bonds (*BO_ij_^π^*), and π-π bonds (*BO_ij_^π-π^*) as Equation (7).

(7) $$\begin{array}{l} {BO}_{ij}^{\prime }=BO_{ij}^{\sigma }+BO_{ij}^{\pi }+BO_{ij}^{\pi \pi }\\ =\exp[p_{bo,1}(\frac{r_{ij}{}^{\sigma }}{r_{o}^{}})p_{bo,2}]+\exp[p_{bo,3}(\frac{r_{ij}{}^{\pi }}{r_{o}^{}})p_{bo,4}]+\exp[p_{bo,5}(\frac{r_{ij}{}^{\pi \pi }}{r_{o}^{}})p_{bo,6}] \end{array}$$

where *r_o_* is the equilibrium distance; *r_ij_*^σ^, *r_ij_*^π^, and *r_ij_*^ππ^ are the interatomic distance for σ-bonds, π-bonds, and π-π bonds, respectively; and *p_bo_*_,1_, *p_bo_*_,2_, *p_bo_*_,3_, *p_bo_*_,4_, *p_bo_*_,5_, and *p_bo_*_,6_ are the fitting parameters. Then, the energy terms can be derived from bond order values; for example, the bond dissociation term *E*_bond_ can be described as Equation (8).

(8) $$E_{bond}=-D_{e}^{\sigma }BO_{{}_{ij}}^{\sigma }\exp[p_{be1}(1-BO_{ij}^{\sigma }{}^{p_{be2}})]-D_{e}^{\pi }BO_{{}_{ij}}^{\pi }-D_{e}^{\pi \pi }BO_{ij}^{\pi \pi }$$

where *D_e_^σ^*, *D_e_^π^*, and *D_e_^ππ^* are the bond parameters for σ-bonds, π-bonds, and π-π bonds, respectively; *p_be_*_1_ and *p_be_*_2_ are the fitting parameters. ReaxFF [98,99,100] was initially developed for simulating combustion of hydrocarbon and reactions of energetic compounds, and the functional groups such as nitro group are well-parameterized, thus various energetic compounds such as RDX, HMX, CL-20, PETN, NM, TNT, TATB, and TATP were applied [115,116,117,118,119,120,121,122,123,124,125,126,127,128,129,130,131,132,133,134,135,136]. The ReaxFF-based MD simulations provide an efficient way to investigate the chemical reactions and other properties of various molecular crystals under particular conditions.

### 4.2. Applications

#### 4.2.1. Structural Optimization

Although ReaxFF has been applied to optimize crystal structures [106,115], it can hardly be comparable to the aforementioned classic FFs, because it is in principle developed for describing reactions. Thus, the structure optimization by ReaxFF is mainly for stress relaxation, instead of structural prediction.

#### 4.2.2. Vibration Spectra

**Figure 4:**
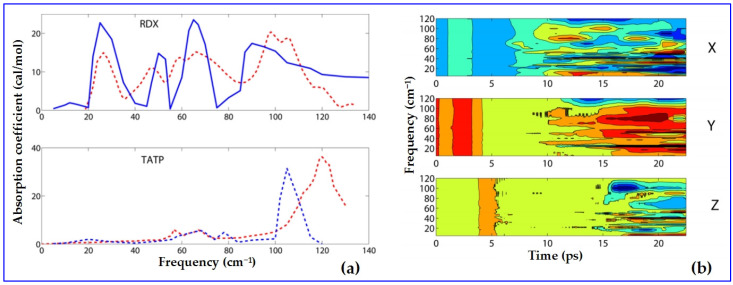
Calculated THZ spectra of RDX and TATP (in blue) vs. experimental (in dashed red) (**a**) and calculated 2D THz spectra of time domain dipole response for different excitation frequencies (**b**). Reprinted by permission from [116]; copyright 2014 American Chemical Society. Permission conveyed through Copyright Clearance Center, Inc.

Vibration property of the energetic crystal can also be predicted using ReaxFF, since it contains the interaction terms for atoms. Katz et al. [116] applied MD simulations on crystalline RDX and TATP to obtain the terahertz absorption and 2D spectroscopy, in which ReaxFF was used and radiation absorption was simulated directly by adding an oscillating electric field to the MD FF. The calculated THz spectra of RDX and TATP are comparable to the experimental observations. Moreover, considering the excitation frequency, the 2D THz spectrum was calculated, showing the time domain dipole response for different excitation frequencies, as well as 2D spectrum as a function of the excitation frequency (Figure 4).

#### 4.2.3. Shock-Induced Chemistry

**Figure 5:**
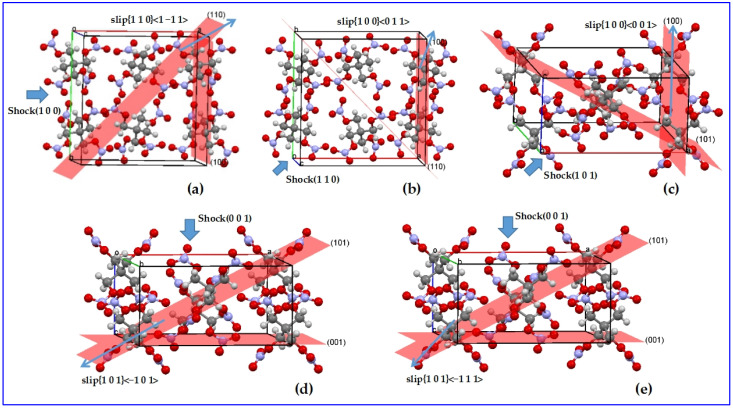
Diagram of CS-RD model for PETN (**a**) shock along (1 0 0) and slip along {1 1 0}<1 −1 1>, represented as (1 0 0)/{1 1 0}<1 −1 1>; (**b**) (1 1 0)/{1 0 0}<0 1 1>; (**c**) (1 0 1)/{1 0 0}<0 0 1>; (**d**) (0 0 1)/{1 0 1}<−1 0 1>; (**e**) (0 0 1)/{1 0 1}<−1 1 1>.

ReaxFF is apt to reveal the initial decomposition and shock induced chemistry for various energetic crystals, such as RDX, TATB, PETN, NM, Si-PETN, and TNT/CL-20 [106,117,118,119,120]. Goddard’s group has developed a ReaxFF-MD-based compress-shear reactive dynamics (CS-RD) procedure (Figure 5) to investigate the anisotropic shock sensitivity of energetic crystals, in which the amount of the chemical reaction products and the kinetics were used to evaluate the sensitivity. The procedure has successfully been applied to energetic molecular crystals PETN, RDX, and HMX; as a result, it was found that the difference in internal molecular steric hindrance contributes to the origin of anisotropic sensitivity of energetic materials [121,122,123,124,125,126]. Special attention also has to be given to the molecular dynamics of insensitive energetic crystal TATB; therein, the shear stress, energy, temperature, and chemical reactions during compress-shear deformation were revealed under a relatively longer loading time, with the shear stress barrier found to be higher than the former energetic crystals [127]. 

Moreover, Strachan et al. [117] used ReaxFF to investigate the shock-induced chemistry for RDX crystal, with the details about shock responses including energetics of unimolecular decomposition mechanisms, shock velocity as a function of particle velocity, as results, time evolution of gas products is revealed, and the composition of gas products is close to that observed experimentally from mass spectra. Wen et al. also used ReaxFF-based MD simulations to compare the initial decomposition of twinned and perfect HMX against shock, showing the faster decomposition of the twinned HMX and, further, the higher shock sensitivity [128]. Xue et al. utilized ReaxFF-MD combined with the multiscale shock technique (MSST) to investigate the evolution of shear stress, temperature, pressure, and gas products for dislocated and perfect RDX crystals under shock, with the edge dislocation proving to be more shock sensitive [129]. Similarly, the coupling effects of shock, heat, and defect were revealed [130]. Moreover, Zhong et al. found the molecular disorder enhanced shockwave-absorption roots for the disorder induced sensitivity enhancement [131].

#### 4.2.4. Thermal Decomposition

Another important aspect of the application of ReaxFF-MD is to disclose thermal decomposition mechanism under various heating conditions, with evolution of the gas products, key reactions, and kinetics detailed [132,133,134,135,136]. ReaxFF-MD has been utilized to compare the thermal reactivity for perfect and imperfect energetic crystals. For example, Deng et al. [132] used ReaxFF-MD to compare the thermal decay of perfect and twinned β-HMX under constant-temperature, programmed, and adiabatic heating conditions, and it was found that enhanced self-heating ability stems from the high internal energy of the molecules around the defects. ReaxFF-MD has also been applied to compare the initial cluster evolution for insensitive TATB, relatively sensitive β-HMX, and sensitive PETN under heating conditions—and a correlation was established between OB and clustering [133,134]. Moreover, it was used to understand the mediated thermal stability of the cocrystal of CL-20/HMX, compared with the pure components [135]. Additionally, it was applied to investigate the effect of volume filling degree (VFD) on the thermal decomposition and detonation of RDX crystal, showing that the higher VFD is more conductive for an explosion [136].

Despite the aforementioned wide use of the ReaxFF-MD, the reliability to new compounds like heterocycles and energetic ionic salts has not been verified sufficiently. In addition, the ReaxFF-MD significantly depends on the elements, it can hardly be applied for a special system with elements uncovered in the FF.

#### 4.2.5. NN-Trained ReaxFF (NNRF)

**Figure 6:**
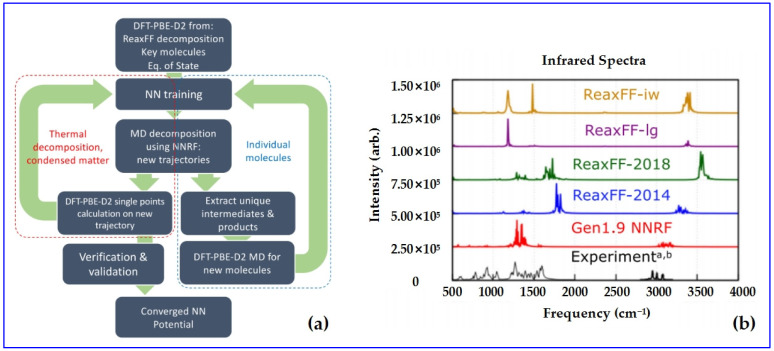
Fitting procedure of NNRF (**a**) and the predicted infrared spectra of RDX using NNRF (**b**). Reprinted by permission from [143]; copyright 2021 Springer Nature Limited. Permission conveyed through Copyright Clearance Center, Inc.

The higher accuracy of reactive FFs is pursued for various molecular crystals, as well as extension to more elements [137]; thus, ReaxFF can be continuously improved using more quantum-mechanics-based calculations [138,139] and even machine learning methods [140,141,142]. Yoo et al. [143] have developed a neural network reactive forcefield (NNRF) for studying the reactivity of RDX crystals under a wide range of temperatures, showing higher accuracy for predicting vibration spectra, thermal decomposition, and reactions (Figure 6).

## 5. Conclusions and Outlooks

The commonly used FF methods for describing energetic molecular crystals—including classic, consistent, and reactive FFs—are reviewed in this article, covering basic principles and prediction applications, such as polymorphism, morphology, thermodynamics, vibration spectra, thermal property, mechanical property, and reactivity. The classic FFs are still useful for typical energetic crystals—i.e., even though they generally have simple potential functions, the accuracy can be ensured by refitting for specific compound. The consistent FFs with more complicated function forms and abundant fitting parameters are much easier to use, and can be considered in coding for high-throughput computations. The reactive FFs are a powerful tool which can effectively describe the reactivity of many energetic crystals under various conditions; in addition, machine-learning procedure is also applicable to improve the accuracy of FFs. 

Compared with AIMD and DFTB-MD simulations, those based on FFs can significantly enlarge simulation size and elongate simulation time, even to the mesoscale. Refitted classic FFs are recommended for the predictions of basic properties of typical energetic crystals, including cell parameters, density, and key material properties as shown in Figure 1, thermal properties prediction using SAPT and NETMFF, polymorph prediction using OPLS-AA, vibration spectra calculation using Bedrov’s FF, and mechanical properties using SRT or SB FFs; on the other hand, the consistent FF COMPASS is suggested for the structural optimization and morphology prediction of various energetic crystals, and other properties—such as thermodynamics, thermal expansion, mechanical properties, and stability—can also be easily predicted using COMPASS without any refitting; moreover, the reactive ReaxFF is feasible for chemical reaction related properties, including shock-induced chemistry and thermal decomposition; for some non-chemical properties such as vibration spectra, ReaxFF is also applicable.

To date, the prediction accuracy of the FF methods also depends on the molecular structure and functional groups of object compounds. For aliphatic energetic compounds, SRT potential was applied on heterocycles RDX, HMX, and CL-20, as well as linear PETN and FOX-7; in which the accuracy is higher for RDX, HMX, and CL-20 with relatively rigid molecules inside the crystal, while the accuracy is lower for nitrate PETN with floppy molecules at higher pressures. SB was appropriate for nitramines RDX, HMX, and CL-20—especially their mechanical responses; and Boyd’s FF or NETMFF were also suitable for RDX. For the aromatic nitro compounds, which have more conjugated structures, GRBF, Bedrov’s, and Dreiding potentials were fit for TATB. Meanwhile, Neyertz’s FF was fit for TNT and DNT. Moreover, COMPASS and ReaxFF—with abundant fitting parameters—are applicable for most nitro energetic compounds including RDX, HMX, CL-20, FOX-7, TATB, and TNT; these FFs differ in the application properties, COMPASS is appropriate for non-chemical properties with relative low accuracy demands, while ReaxFF is appropriate for decomposition or detonation properties related to chemical reactions; in addition, for nitrate ester PETN or Si-PETN with large substituents, ReaxFF is also well-applied.

It should also be noted that, since empirically derived FFs are adapted to a finite set of equilibrium situations, and mostly experimental data and ab initio results are for equilibrium configurations, they cannot transfer to different excitation situations or situations relying quantitatively on detailed electronic structures. Moreover, the accuracy of FF methods highly depends upon the quality of the molecular FFs. Many new FFs are highly desired for new systems and new issues. Nowadays, there are still large computational demands for describing structural, electronic, and dynamic properties of large and complex energetic crystals with technologically relevant size, simulation time, and accurate solutions. It is important to select appropriate methods, such as refitted FFs, for dealing with energetic molecular crystals efficiently and precisely—as well as efficient methods such as the consistent FFs for designing programs with high-throughput computations of EMs.

## Data Availability

All reported or analyzed data in this review are extracted from published articles.

## Abbreviations

BTF:	benzotrifuroxan
CL-20:	2,4,6,8,10,12-hexanitro-2,4,6,8,10,12-hexaazaisowurtzitane
DATNBI:	4,4′,5,5′-tetranitro-1H,1′H-[2,2′-bi-imidazole]-1,1′-diamine
DNAN:	2,4-dinitroanisole
DNP:	3,4-dinitro-1H-pyrazole
DNT:	2,4-dinitrotoluene
FOX-7:	1,1-diamino-2,2-dinitroethene
HMX:	octahydro-1,3,5,7-tetranitro-1,3,5,7-tetrazocine
MATNB:	1-methyl-amino-2,4,6-trinitrobenzene
MTNP:	1-methyl-3,4,5-trinitro-1H-pyrazole
MTO:	2,4,6-triamino-1,3,5-triazine-1,3,5-trioxide
MTO3N:	2,4,6-trinitro-1,3,5-triazine-1,3,5-trioxide
NM:	nitromethane
PETN:	2,2-bis[(nitrooxy)methyl]propane-1,3-diyldinitrate
RDX:	1,3,5-trinitro-1,3,5-triazinane
TATB:	1,3,5-triamino-2,4,6-trinitrobenzene
TATP:	triacetone triperoxide
TNA:	2,4,6-trinitroaniline
TNAZ:	1,3,3-trinitroazetidine
TNB:	1,3,5-trinitrobenzene
TNT:	1,3,5-trinitrotoluene
